# The Effect of Sleep Deprivation on Brain Fingerprint Stability: A Magnetoencephalography Validation Study

**DOI:** 10.3390/s24072301

**Published:** 2024-04-04

**Authors:** Michele Ambrosanio, Emahnuel Troisi Lopez, Arianna Polverino, Roberta Minino, Lorenzo Cipriano, Antonio Vettoliere, Carmine Granata, Laura Mandolesi, Giuseppe Curcio, Giuseppe Sorrentino, Pierpaolo Sorrentino

**Affiliations:** 1 Department of Economics, Law, Cybersecurity and Sports Sciences (DiSEGIM), University of Naples “Parthenope”, 80035 Nola, Italy; 2 Institute of Applied Sciences and Intelligent Systems, National Research Council, 80078 Pozzuoli, Italy; 3 Institute of Diagnosis and Treatment Hermitage Capodimonte, 80145 Naples, Italy; 4 Department of Medical, Movement and Wellness Sciences (DiSMMEB), University of Naples “Parthenope”, 80133 Naples, Italy; 5 Department of Humanities, University of Naples Federico II, 80133 Naples, Italy; 6 Department of Biotechnological and Applied Clinical Sciences, University of L’Aquila, 67100 L’Aquila, Italy; 7 Institut de Neurosciences des Systèmes, Aix-Marseille Université, 13005 Marseille, France; 8 Department of Biomedical Sciences, University of Sassari, 07100 Sassari, Italy

**Keywords:** magnetoencephalography, brain fingerprint, sleep deprivation, functional connectome, brain network

## Abstract

This study examined the stability of the functional connectome (FC) over time using fingerprint analysis in healthy subjects. Additionally, it investigated how a specific stressor, namely sleep deprivation, affects individuals’ differentiation. To this aim, 23 healthy young adults underwent magnetoencephalography (MEG) recording at three equally spaced time points within 24 h: 9 a.m., 9 p.m., and 9 a.m. of the following day after a night of sleep deprivation. The findings indicate that the differentiation was stable from morning to evening in all frequency bands, except in the delta band. However, after a night of sleep deprivation, the stability of the FCs was reduced. Consistent with this observation, the reduced differentiation following sleep deprivation was found to be negatively correlated with the effort perceived by participants in completing the cognitive task during sleep deprivation. This correlation suggests that individuals with less stable connectomes following sleep deprivation experienced greater difficulty in performing cognitive tasks, reflecting increased effort.

## 1. Introduction

In recent years, the so-called “functional connectome” (FC) has been widely employed in studying brain functions both in health and disease [1]. The FC is represented as a matrix in which each component quantifies the statistical interdependency between pairs of brain regions disposed on the corresponding rows and columns. The patterns expressed by FC have been mainly explored by comparing different populations or groups of individuals in different conditions. More recently, the enhancement of tailored therapies has led to interest in a deeper understanding of the potential information contained in a subject-specific FC (i.e., the brain fingerprint). Within this framework, Amico and Goñi developed the concept of the “identifiability matrix”, a mathematical approach able to differentiate different subjects on the basis of the FC [2]. The identifiability matrix is defined as the Pearson correlation between the test and retest FCs of individuals within a specific dataset, allowing one to assess whether two FCs of the same individual are more similar than two FCs of different individuals.

This approach has demonstrated its effectiveness in the clinical field; in fact, the loss of brain fingerprinting predicts clinical impairment in several neurological diseases, such as Alzheimer’s disease [3]. However, it is worth noting that the fingerprint performance is highly dependent on the specific experimental conditions [4]. The usual procedure for fingerprint evaluation considers consecutive acquisitions spaced by a few seconds/minutes, and thus, the quest for identifiability is fulfilled in a limited time interval, leading to a paramount question regarding its stability across time [5]. Investigations dealing with the relationship between brain fingerprints and time are still open, and several works have explored this aspect, considering different time intervals spanning from a few weeks to 1–2 years [5,6,7,8,9]. In these works, it is possible to observe a stable pattern of the fingerprint across time, with some exceptions in cases of neuropsychiatric disorders, such as in subjects with schizophrenia spectrum disorders and in individuals with a high cumulative polygenic risk for schizophrenia [8]. The aforementioned considerations arouse a debate on the impact that some non-physiological conditions can have on the stability of the fingerprint across time.

Another poorly investigated aspect concerns the stability of the fingerprint in relation to stress conditions [4]. Among the possible stress conditions, sleep deprivation represents an interesting case study since it has been shown to alter the brain network [10,11] in a temporary and non-pathological fashion, allowing one to observe possible individual variations. Even though the underlying neurophysiological mechanisms are poorly understood, several studies have investigated dynamic changes in brain connectivity, including electroencephalography (EEG) and functional magnetic resonance imaging (fMRI) approaches [12,13,14], which underline severe alterations in the connectivity of several networks, including dorsal attention, default mode, and hippocampal networks. 

In this framework, the scope of the present study is threefold. Firstly, we aimed to investigate the stability of the FC fingerprint over time. Most of the works that deal with the stability of the fingerprint compare recordings occurring at two different time instants very close to each other (e.g., a few minutes). In this manuscript, we explored the stability of the brain fingerprint by considering two recordings far from each other in time, i.e., measures collected at different times of day. Secondly, we aimed to verify whether stressful conditions such as sleep deprivation can interfere with the stability of the connectome fingerprint. Lastly, we aimed to explore the correlation between reduced stability in the brain fingerprint and perceived stress in performing a cognitive task.

For this purpose, we analyzed data from twenty-three young males in three different MEG recording sessions performed at three different time points. MEG allows for data acquisition with considerable temporal and spatial resolution [15]. Additionally, we co-registered the MRI scans of individual participants to enable an accurate reconstruction of signal sources. The first session was performed in the morning after a night of usual sleep, the second in the evening after twelve hours, and, finally, the third the following morning, after a night spent in the company of the other participants. During each session, two distinct recordings separated by a few minutes were carried out to obtain the FC fingerprints of each participant and to compare them between the three different time points. After filtering the source-reconstructed signals in the five canonical frequency bands, we used the phase linearity measurement (PLM) to build the FCs of each participant [16]. Finally, in order to verify whether the possible reduction in brain fingerprint identifiability would relate to cognitive impairment or sleepiness, after each session, cognitive performance (selective attention and switching ability) and the subjective level of sleepiness were assessed. 

## 2. Materials and Methods

### 2.1. Participants

Thirty-two young male adults were enrolled (mean age ± standard deviation, 24.84 ± 2.85 years), but nine were excluded from the analysis, as further explained in the Statistical Analysis section (i.e., new sample size = twenty-three participants). Female participants were not included in the study design due to the hormonal variations in the menstrual cycle, which influence brain connectivity [17]. All the participants were right-handed Italian speakers, and none of them had any history of medical, neurological, or psychiatric illness nor medication or drug intake. The requirements for the involvement in the experiment were normal sleep duration and no excessive daytime sleepiness. The ingestion of coffee, beverages containing stimulating active ingredients, and intense physical activity were prohibited starting 24 h before the experimental procedure, which was performed during the working week to avoid changes related to weekend activities. All the participants gave their written informed consent. The study was approved by the Ethical Committee of Psychological Research of the Department of Humanities of the University of Naples Federico II (prot. n. 11/2020) and was conducted in accordance with the Declaration of Helsinki.

Concerning sleep quality, a proper qualitative and quantitative analysis on the participants was performed via the Pittsburgh sleep quality index (PSQI) [18], the Epworth sleepiness scale (ESS) [19], and the Karolinska sleep diary (KSD) [20]. The thresholds to allow the subjects to take part in the study were fixed at 5 for the PSQI and 10 for the ESS; thus, participants with scores lower than the previously mentioned thresholds were allowed to take part in the study.

### 2.2. Sleep Deprivation Protocol (SDP)

The procedure involved groups of four participants per night of the SDP. The experimental protocol included three sessions which took place at 9.00 a.m. on day 1 ($M_{1}$), 9.00 p.m. on day 1 $(E_{1}$), and 9.00 a.m. on day 2 ($M_{2}$). In each session, the participants underwent two consecutive MEG recordings at rest separated by 1 min. Immediately after, the subjects performed a letter cancellation task (LCT) and task switching (TS) test (see in the Cognitive Assessment section for more info on these tasks). During each experimental session, the participants were seated on a comfortable chair in a soundproof room. After the first session, the participants were free to return to their daily life activities and then come back to the laboratory for the next session ($E_{1}$) in the evening and start the SDP under the experimenter’s supervision. Short walks outside the laboratory were allowed to prevent the participants from falling asleep. In all the sessions, the perceived subjective state of sleepiness was assessed through the administration of the Karolinska sleepiness scale (KSS) [21] and the cognitive load by means of the NASA Task Load Index (NASA-TLX). In more detail, the NASA-TLX is a multidimensional scale designed to obtain the cost incurred by an individual to achieve a particular level of performance while performing a task. It consists of six subscales that refer to mental (RM), physical (RF), and temporal (RT) demands; effort (S); performance (P); and frustration (F) [22,23]. The NASA-TLX test is considered the most cited and widely used test for workload assessment [24]. It is widely applicable across various contexts and fields of research due to its versatility and generalizability. Its items are easily adaptable, making it suitable for diverse scenarios and allowing researchers to assess cognitive workload effectively [25].

### 2.3. MRI Acquisition

All the participants were subjected to magnetic resonance imaging (MRI) after the SDP, then after the MEG recording in order to minimize the noise derived from electromagnetic fields. These data were fundamental for allowing an accurate source reconstruction with regard to the MEG signal processing pipeline. The MRI images of thirty-two young male adults were acquired on a 1.5 T Signa Explorer scanner equipped with an 8-channel parallel head coil (General Electric Healthcare, Milwaukee, WI, USA). In particular, three-dimensional T1-weighted images were acquired (details are reported in [26]).

### 2.4. MEG System

**Figure 1:**
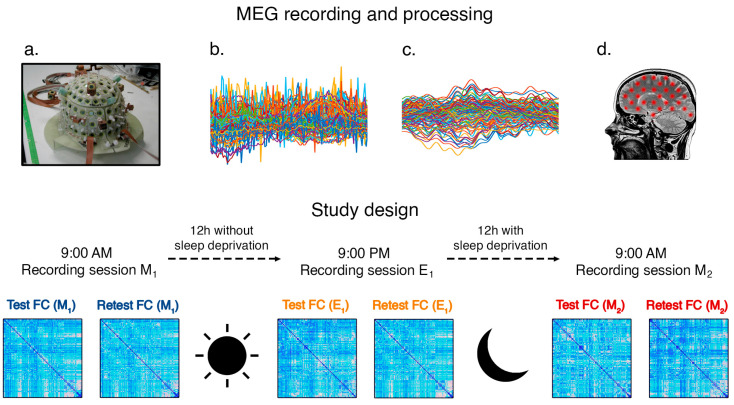
Details of the MEG recording and data processing pipeline. The neuronal activity was recorded via 154 SQUID sensors (**a**); then, the raw signal was filtered from the noise (e.g., cardiac activity and blinking artifacts) (**b**,**c**). The cleaned signal was then co-registered with the MRI signal of each subject to obtain the source activity reconstruction (**d**). Finally, the functional connectivity among brain areas was estimated for each of the 90 brain areas, obtaining a functional connectome (FC) for each frequency band. Two recordings (test and retest) were performed in three sessions (first morning (M1), evening (E1), and second morning (M2)).

The data were acquired by using a MEG system equipped with SQUID magnetometers developed by the Institute of Applied Sciences and Intelligent Systems at the Italian National Research Council [27]. The MEG system consisted of an ultra-thermally insulated cylindrical container (dewar), inside which the SQUID sensors were placed on a helmet-shaped support to adapt to the shape of the patient’s head (Figure 1a); the dewar was filled with liquid helium (T = 4.2 K) to cool the sensors to its working temperature. The distance between neighboring sensors was 3 cm, while the distance from outside the dewar, where the patient’s head was housed, was just 2 cm thanks to the effectiveness of thermal insulation, which ensured a normal room temperature on the external surface of the dewar. The helmet included 154 SQUID magnetometers as measurement channels, while another 9 SQUIDs were organized into three triplets and positioned further away from the measurement surface to measure the environmental noise. 

Such sensors consist of fully integrated SQUID magnetometers based on a Ketchen-type design and include a superconducting flux transformer inductively coupled to the SQUID loop in a washer shape [28]. Since the readout electronics require a negative feedback circuit to linearize the output extending the linear dynamic range, the magnetometer also included a feedback coil in a bipolar shape to minimize the crosstalk between the neighboring channels. All coils and a resistor network for the SQUID operation were integrated on the same chip in order to avoid additional noise due to external circuit elements. The SQUID sensitivity, usually reported as the spectral density of magnetic field noise, measured at T = 4.2 K was less than 2.0 fT/√Hz down to 1–2 Hz.

In order to drastically reduce the environmental magnetic signals, which are much more intense than the signals generated by the brain, the system and the patients were housed in a magnetic shielded room made of a layer of aluminum and two layers of μ-metal (high-permeability materials), showing a shielding factor of 35 dB at 10 mHz that increases up to 100 dB starting from 20 Hz. The background residual magnetic noise inside the shielded room was about 5 fT/√Hz, which represents the sensitivity of the MEG system [27]. Note that the noise level does not depend on the signal amplitude, which arises only from the brain activity. After the first system cooling, each sensor was set at the optimal working point and related values were stored in a configuration file. The sensors always remained at T = 4.2 K, with the liquid helium refilled periodically (one time per week). The configuration file was recalled if the sensors were turned off in order to reduce the liquid helium consumption. 

Furthermore, it should be noted that unlike electric fields, magnetic fields recorded by SQUIDs are less prone to distortion by the scalp and skull [29].

### 2.5. MEG Acquisition and Processing

MEG data were acquired and processed like in [30]. In particular, two closed-eye resting-state segments were recorded, each lasting 3.5 min, with a minute interval between the two consecutive acquisitions. During the acquisitions, the volunteers were seated inside a magnetically shielded room. Moreover, before the measurements, the position of four anatomical landmarks (nasion, right and left pre-auricular points, and vertex of the head) and the position of four reference coils (attached to the head of the subject) were digitalized by using Fastrak (Polhemus^®^, FTGui v1.0.0.1, Colchester, VT, USA) to define the position of the head under the helmet. Before each segment of the registration, the position of the head was checked, and during the acquisition, cardiac activity and eye blinking were recorded by using an electrocardiogram and electro-oculogram, respectively, to remove physiological artifacts. After that, an anti-aliasing filter was applied, and the data were sampled at 1024 Hz. A detailed description of the processing pipeline is available in the Supplementary Materials.

Consequently, the MEG data were filtered in the frequency band of interest (0.5–48 Hz) using a 4th-order Butterworth IIR band-pass filter implemented using Matlab scripts in the Fieldtrip toolbox [31]. As reported in [26], the data were then processed via principal component analysis (PCA) for environmental noise reduction, and noise channels and bad segments of the acquisition were successively identified and removed via visual inspection by an experienced rater, like in [32]. Then, the signal was processed via independent component analysis (ICA) and visual inspection for physiological-artifact reduction (e.g., eye blinking and heart activity). 

A linearly constrained minimum variance (LCMV) beamformer [33] was adopted to reconstruct the time series signals related to the centroids of 116 brain regions of interest (ROIs), according to the Automated Anatomical Labelling atlas. To this aim, the geometrical information derived from the MRI acquisition and the volume conduction model proposed by Nolte [34] were exploited. Lastly, the reconstructed time series were filtered into five standard frequency bands: delta (0.5–4.0 Hz), theta (4.0–8.0 Hz), alpha (8.0–13.0 Hz), beta (13.0–30.0 Hz), and gamma (30.0–48.0 Hz). Figure 1 illustrates the whole MEG data processing pipeline.

To provide an estimate of the connectivity among each pair of brain areas, a phase linearity measurement (PLM) was performed [16]. In particular, the 26 cerebellar regions were excluded from the analysis due to the low reliability of the signal, and thus, 90 regions encompassing the cerebral cortex and the basal ganglia were considered. The result of this operation was the generation of a 90 × 90 matrix describing the connectivity between brain areas for a specific subject (also known as the “functional connectome” (FC)). To perform the analysis, we constructed a matrix for each of the two recording trials in each session. Thus, we obtained one FC test and one FC retest for each of the three sessions for each subject [2,4,35].

### 2.6. Fingerprint Analysis

As mentioned in the previous subsection, fingerprint analysis based on FCs is a methodology able to define subject-specific characteristics. Briefly, it starts from the definition of a matrix known as an “identifiability” or “differentiation” matrix [2]. In this context, the similarity is defined as the Pearson correlation between the FCs at hand. Thus, the identifiability matrix “A”, i.e., the matrix of correlations (square and non-symmetric) between the subjects’ FCs’ test and retest, has subjects both as rows and columns and encodes information regarding the similarity of each subject with themself (the main diagonal of the matrix—*I_self_* metric in Formula (1)), as well as the similarity of each subject to the others (off-diagonal elements—*I_others_* metric in Formula (2)). Thus, starting from the identifiability matrix “A”, the following metrics can be defined [2]:

(1) $$I_{self}^{i}{=a}_{ii}$$

(2) $$I_{others}^{i}=\frac{1}{2N}\underset{\;\;j\neq i}{\sum_{j=1}^{N}}\left(a_{ij}+a_{ji}\right)$$

(3) $$I_{diff}^{i}=I_{self}^{i}-I_{others}^{i}$$

(4) i=1,2,...,N

in which “$a_{ij}$” refers to the element of the identifiability matrix at the i-th row and j-th column, and “N” is the number of subjects. It is worth noting that the metric *I_diff_* quantifies the difference between the average within-subject-FC similarity and the between-subject-FC similarity. The higher this value, the higher the individual fingerprint overall across the population.

More specifically, we considered four different combinations to perform the analysis and verify the stability of fingerprint analysis as a function of time and stress (i.e., one night of sleep deprivation):

- Combination M_1_M_1_: this refers to the test and retest collected on the same day at very close time instants during the morning (one-minute time distance)—no sleep deprivation.
- Combination M_1_E_1_: this refers to the comparison between the morning acquisitions and the evening acquisitions—no sleep deprivation.
- Combination E_1_M_2_: this refers to the comparison between the evening acquisitions and the next-morning acquisitions—including sleep deprivation.
- Combination M_1_M_2_: this refers to the comparison between the two different morning acquisitions—including sleep deprivation.

In each case where different sessions were compared, the fingerprint was calculated by averaging the comparison between the test of session 1 and the retest of session 2, and vice versa.

### 2.7. Cognitive Assessment

After each MEG registration, the participants performed the cognitive assessment tests via LCT and TS. For each test, the experimenter provided the instructions and left the room immediately, making sure that the tests ran without any distraction in the soundproof room. 

#### 2.7.1. Letter Cancellation Task

The letter cancellation task [36] required participants to sequentially search and mark (from left to right and from top to bottom), as fast and accurate as possible, three target letters within a 36 × 50 matrix of capital letters (font: New York Times, “12”) printed on an A4 paper sheet. The total time allowed for the completion was 5 min, and every target appeared 100 times in a random sequence. The number of hits, assumed as a measure of accuracy, and the number of completed rows, assumed as a measure of speed, were considered dependent variables.

#### 2.7.2. Task Switching

In task switching, two different tasks were considered in rapid succession according to the presentation of a random sequence of tasks. Thus, two consecutive tasks might represent the same one, i.e., a “repetition” trial, or different ones, i.e., a “switch” trial. In this specific experiment, the two tasks consisted of the following: (i) task A: the digit is odd or even; (ii) task B: the digit is greater or smaller than 5.

The difference in terms of accuracy and time for the two trials was referred to as “switch cost”, which can be considered an operational measure of executive control [37]. All the participants were individually tested in a comfortable, soundproof room and sat in front of a 15-inch monitor illustrating the tasks. The instructions were both displayed on the screen and explained verbally by the experimenter at the beginning of each session, underlining the need for both accuracy and speed in performing the TS test. Further details on how the TS test was performed can be found in [26].

### 2.8. Subjective Evaluations

Two subjective sleepiness evaluations were used in the protocol: the Karolinska sleepiness scale (KSS) and the NASA Task Load Index (NASA-TLX) [23]. The former scale measures the subjective level of sleepiness at a particular time during the day. The KSS is a measure of situational sleepiness, and it is sensitive to fluctuations. It is a 10-point scale and self-report measure which takes 5 min to be completed. The latter, the NASA-TLX, is a subjective assessment tool used to measure the perceived workload and mental demands experienced by individuals while performing tasks. This test assesses the workload across six dimensions: (i) mental demand (RM): the cognitive effort and complexity required to perform the task.; (ii) physical demand (RF): the physical effort and exertion required to complete the task, (iii) temporal demand (RT): the time pressure or urgency associated with the task; (iv) performance (P): the perceived level of success or accomplishment in performing the task, (v) effort (S): the level of effort or exertion invested in completing the task; (vi) frustration (F): the extent to which the task was frustrating, stressful, or challenging.

For each dimension, participants rated their perceived workload on a scale ranging from low to high. The ratings were then combined using a weighting and averaging procedure to calculate an overall workload score, which indicates the perceived workload intensity.

### 2.9. Statistical Analysis

To identify potential significance among the different experimental conditions, a PERMANOVA test (with 10,000 permutations) was run on the four different combinations (i.e., M_1_M_1_, M_1_E_1_, M_1_M_2_, E_1_M_2_) for each metric described in the Fingerprint Analysis section and for each frequency band (i.e., delta, theta, alpha, beta, and gamma). After that, a post hoc analysis via a permutation test was performed to identify the pairs that significantly differed. Specifically, the labels of the two given cases were shuffled 10,000 times to obtain two surrogate groups of values, and each time, the absolute difference of the mean values of the two surrogate groups was computed. Finally, the absolute difference of the actual cases was compared to the 10,000 surrogate differences to obtain a statistical significance. Lastly, the results were corrected by adopting the Benjamini–Hochberg procedure (BH step-up procedure) [38] to control the false discovery rate (FDR) at a level of 0.05. 

Subsequently, Pearson’s correlation was used to find possible correlations between fingerprint metrics and behavioral performance. A correlation analysis was carried out between the fingerprint metrics and the difference between the tests’ scores (Δ) corresponding to sessions used to estimate the fingerprint metrics. For instance, the *I_self_* related to the M_1_E_2_ case was correlated with the difference between the cognitive scores obtained during the first morning and the second morning. These tests were conducted on a population of 23 subjects, since 9 subjects were excluded from the analysis as it was not possible to obtain both test and retest connectomes for each recording session, following the signal cleaning procedure.

## 3. Results

**Figure 2:**
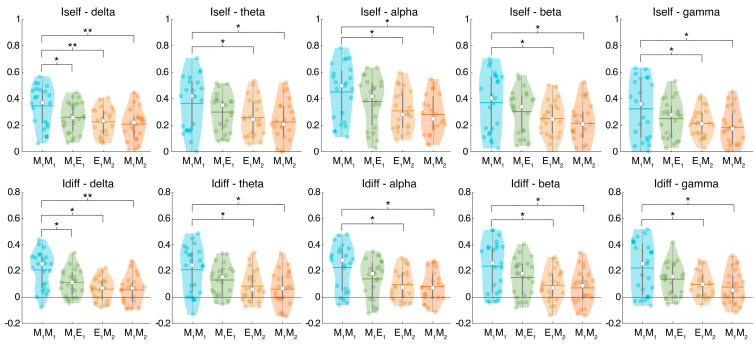
*I_self_* and *I_diff_* for each frequency band. Violin plot of the fingerprint scores representing the similarity between two functional connectomes (FCs) of the same individual obtained at different time points (*I_self_*), and the extent to which an individual is differentiable within the dataset considering each case (*I_diff_*). Colored dots represent the individuals, horizontal lines represent the average value, and white dots represent the median value. M_1_M_1_ compares the two recordings performed during the first morning (9:00 a.m. on day 1)—1 min distance; M_1_E_1_ compares the FC of the first morning and the FC of the evening (9:00 p.m. on day one)—12 h distance; E_1_M_2_ compares the FC of the evening and the FC of the second morning (9:00 a.m. on day two)—12 h distance including sleep deprivation; M_1_M_2_ compares the FC of the first morning and FC of the second morning—24 h distance including sleep deprivation. * = *p_fdr_* < 0.05, ** = *p_fdr_* < 0.01.

We found a statistically significant difference in all frequency bands for both *I_self_* and *I_diff_* when the basal combination (i.e., M_1_M_1_) was compared with the other three combinations (i.e., M_1_E_1_, E_1_M_2_, and M_1_M_2_). In particular, the values of *I_self_* and *I_diff_* obtained from the fingerprinting of the first morning session were higher than (1) the fingerprint between the evening and the second morning, and (2) the fingerprint between the first and the second mornings. It should be noted that both comparisons included a night of sleep deprivation and only differed in the time passing between the sessions. Interestingly, the *I_self_* and *I_diff_* values of the basal condition (i.e., first-morning recordings) were not significantly different from the *I_self_* and *I_diff_* values of the combination without sleep deprivation (i.e., the M_1_E_1_), except for the delta band. Figure 2 shows the comparison for the *I_self_* metric as well as the *I_diff_* metric.

**Figure 3:**
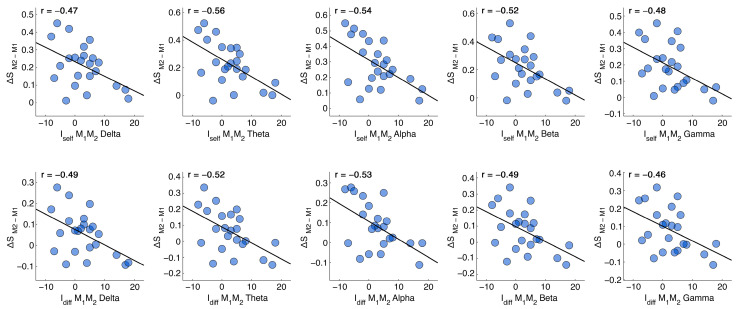
Pearson’s correlation analysis of Iself and Idiff with ∆S of the NASA-TLX in the five frequency bands for the M_1_M_2_ case (24 h with sleep deprivation). The difference in perceived effort is negatively correlated in each frequency band, hence, the higher the effort, the lower the differentiation of the subject.

After the identification of the significant differences between the four combinations, a correlation analysis (Pearson’s correlation) was carried out. In detail, we performed a correlation test between the *I_self_* and *I_diff_* fingerprint metrics of the groups M_1_M_2_ and E_1_M_2_ (the ones presenting significant differences across all frequency bands) and the differences in scores within the respective time points on the cognitive tests (LCT, TS), subjective sleepiness evaluations (KSS), and stress perceived in performing the cognitive tests (NASA-TLX). Specifically, the notation “∆X” refers to the difference between the specific “X” subscore of the NASA-TLX test between two sessions. For instance, if the subscore “S” (effort) is considered between the times $M_{1}$ and $M_{2}$, then the adopted notation will be $∆S=S_{M_{1}}-S_{M_{2}}$. Following FDR correction, we found that the *I_self_* between the two morning recordings was inversely correlated with the ΔS in all frequency bands. Hence, the higher the similarity of the recordings after 24 h including sleep deprivation, the lower the perceived effort in performing cognitive tasks. The *I_diff_* presented the same significant inverse correlations, suggesting that the more an individual was differentiable within the dataset, the lower the effort was in completing the cognitive tasks. None of the remaining tests reported significant correlations. Statistical data are reported in Table 1, while the scatter plots of the correlations are reported in Figure 3.

## 4. Discussion

In this work, we explored the stability of the FC over a 24 h period, assessing the ability to differentiate each individual from all the others. Moreover, we also evaluated the effect of a particular stress condition, i.e., sleep deprivation, on the stability of the brain fingerprint. Finally, we investigated the possible correlation between the brain fingerprinting characteristics, cognitive condition, and subjective sleepiness evaluations assessed before and after sleep deprivation. To this aim, a population of 23 healthy young adults was tested. Based on the source-reconstructed MEG signals, test and retest FCs were generated for each recording session carried out 12 h apart, with the second and third recording sessions interspersed with a night of sleep deprivation. The first result concerns the stability of the connectomes at a 12 h time distance. The comparison between *I_self_* values in all frequency bands, except the delta band, obtained from the test and retest recordings performed at 9 a.m. on the first day and 9 p.m. on the same day was not statistically different from the comparison of recordings that occurred only in the early morning. This also applies to the differential score (*I_diff_*), which, besides reaffirming the similarity between the recordings performed at different times, also confirms fair discrimination among the different participants included in the dataset. These results demonstrate the stability of the FC fingerprint over time (at least limited to 12 h of daytime).

Such an aspect has been investigated by several articles using different technologies (e.g., fMRI, EEG, intra-cranial electrodes, etc.), but almost no work has explored this aspect using MEG, which represents one of the main novel aspects of the proposed analysis. One study, by adopting the fMRI dataset from the Human Connectome Project (www.humanconnectome.org, accessed on 1 April 2024), found that the best identification of brain fingerprints occurs at longer time scales, but short bursts of identifiability associated with neuronal activity persist even at shorter time scales [39]. Additionally, Ousdal et al., investigating the stability of the connectome by means of fMRI, reported that it remains stable over a 2–3-year period in middle and older age [40]. These findings suggest that the brain functional connectome can be stable over time, and our results align with this outcome. It is important to note that in the delta band, a significant decrease in stability after 12 h was observed. The high amplitude of the signals in this frequency band is often associated with deep sleep [41], but numerous studies report that this band also plays a role in the wakeful state, such as in the case of neocortical background activity, memory consolidations, and plasticity modulations [42,43]. Activity in the delta band has also been found to be altered due to sleep inertia effects [44,45] that may also last hours [46]. This may explain why we found such instability in the delta band, even though specific study designs are needed to fully confirm this statement.

Then, we investigated what happens to connectome stability when introducing a stress factor. We observed that the stability of the FC fingerprint over time was reduced following sleep deprivation. In particular, we found a reduction in both the *I_self_* and the *I_diff_* when considering the recordings that occurred 12 (E_1_M_2_) and 24 (M_1_M_2_) hours apart. A reduction in these parameters suggests that the differentiability of individuals decreases, which could inferentially be translated as an alteration of the network occurring following sleep deprivation. Specifically, for the *I_self_*, a reduction in its value indicates less similarity between the two recordings, while there are deeper considerations to be made regarding *I_diff_*, which refers to the differentiability of an individual compared to a group. Hence, considering that differential scores higher than zero represent a positive differentiation of a given subject compared to the group, we can assert that, despite a significant decrease, most participants retained a positive value even after the sleep deprivation night. This suggests that the alteration of the functional network, although capable of altering test–retest repeatability following stressful factors (in this case, sleep deprivation), does not damage the brain network to the extent of altering important subject-specific elements that allow for differentiation. Furthermore, it must be noted that if no significant difference is detected in the *I_others_* scores, the variation in *I_diff_* largely depends upon the reduction in *I_self_*. Several studies have demonstrated the profound impact of sleep deprivation on both structural and functional aspects of the brain. Wang L. et al., using electron microscopic analyses of neurons, reported that sleep loss can alter the structures of various organelles in the brain, which may disrupt fundamental cellular processes [47]. Wang C. et al., using surface morphological analysis and graph theoretical analysis, showed that sleep restriction decreases cortical thickness and enhances the topological properties of the structural covariance network [43]. Additionally, total sleep deprivation affects functional connectivity, as observed by EEG recordings [48] and event-related potential analysis [49]. However, the impairment induced by sleep deprivation appears to be temporary and reversible with sufficient sleep, as demonstrated by resting-state functional MRI data [50].

Another interesting aspect concerns the correlation between reduced individual differentiability (for both the *I_self_* and *I_diff_* parameters) due to sleep deprivation and cognitive assessment. We observed an inverse correlation between *I_self_* and *I_diff_* related to the two morning recordings (M_1_M_2_) and the subscore (effort) of the NASA-TLX test. In other words, we showed that the increase in perceived effort during test performance following sleep deprivation was correlated with reduced stability in the functional connectome. Hence, the subjects who reported having to spend more effort to complete cognitive tasks after a night of deprivation were also those whose connectome had varied the most. Sleep deprivation has been shown to be associated with decreased cognitive performance [51], and changes in functional connectivity following sleep deprivation have been found to correlate with worsened executive functions [52]. Speculatively, we suppose that the augmented effort may be caused by the increased attentional processing required to complete the tasks in hindering conditions. Indeed, sleep deprivation has been found to have a negative impact on attention [53,54]. However, further studies specifically designed to investigate this are required in order to confirm this hypothesis [54].

The described study has several limitations. Firstly, the size of the sample was relatively small and further investigation should focus on larger samples to confirm these results. During the nocturnal period, the participants stayed in groups of four in a lit environment and were allowed to converse with each other. An operator checked that they did not fall asleep. The fact that the control was not performed using a video recording system and that the participants were not alone could represent limitations of the work.

## 5. Conclusions

In conclusion, this study assessed the short-term (24 h) stability of the functional connectome using brain fingerprinting. We observed that a stress-inducing physiological condition can globally impair this temporal stability. Additionally, we found a correlation between impaired fingerprint stability and the perceived effort of participants during cognitive tasks under conditions of sleep deprivation. Furthermore, it is paramount to note that the proposed work represents one of the first studies analyzing the stability of the FC fingerprint over time with MEG data processing, which has been poorly investigated in the scientific literature and which does represent an interesting alternative to conventional technologies (e.g., fMRI, EEG, etc.) thanks to its excellent time resolution.

## Figures and Tables

**Table 1:** Pearson’s correlations (corr) and *p*-values before (*p*-value) and after (*p*-value (FDR)) correction (Benjamini–Hochberg) between the fingerprint metrics $I_{self}$ and $I_{diff}$ and the differences in the S subscore (effort) of the NASA-TLX test for the M_1_M_2_ case for each frequency band.

M_1_M_2_	$∆S-I_{self}$					$∆S-I_{diff}$				
	δ	Θ	α	β	γ	δ	θ	α	β	γ
*p*-value	0.024	0.005	0.008	0.011	0.019	0.017	0.010	0.010	0.017	0.026
*p*-value (FDR)	0.024	0.018	0.018	0.018	0.024	0.022	0.022	0.022	0.022	0.026
corr	−0.47	−0.56	−0.54	−0.52	−0.48	−0.49	−0.52	−0.53	−0.49	−0.46

## Data Availability

The data presented in this study are available on request from the corresponding author (Pierpaolo Sorrentino) upon approval by the ethics committee. Availability for the data was not previously included in the ethical approval, and therefore, data cannot be shared directly. In case the data are requested, the corresponding author will request an amendment from the local ethics committee.

## Supplementary Materials

The following supporting information can be downloaded at: https://www.mdpi.com/article/10.3390/s24072301/s1, Detailed processing and analysis pipeline.
