# Supplementary material for: The Effect of Sleep Deprivation on Brain Fingerprint Stability: A Magnetoencephalography Validation Study
Source: Sensors (Basel). 2024 Apr 4;24(7):2301. doi: 10.3390/s24072301 (PMC11014248; doi:10.3390/s24072301)
Supplement: Supplementary file 1 [file sensors-24-02301-s001.zip › sensors-2927740-supplementary.pdf]

sensors

Supplementary Materials for

The Effect of Sleep Deprivation on Brain Fingerprint Stability: A Magnetoencephalography Validation Study

Michele Ambrosanio [1,†], Emahnuel Troisi Lopez [2,†], Arianna Polverino [3], Roberta Minino [4], Lorenzo Cipriano [4], Antonio Vettoliere [2], Carmine Granata [2], Laura Mandolesi [5], Giuseppe Curcio [6], Giuseppe Sorrentino [2,3,4] and Pierpaolo Sorrentino [2,7,8,*]

[1] Department of Economics, Law, Cybersecurity and Sports Sciences (DiSEGIM), University of Naples "Parthenope", 80035 Nola, Italy
[2] Institute of Applied Sciences and Intelligent Systems, National Research Council, 80078 Pozzuoli, Italy
[3] Institute of Diagnosis and Treatment Hermitage Capodimonte, 80145 Naples, Italy
[4] Department of Medical, Movement and Wellness Sciences (DiSMMEB), University of Naples "Parthenope", 80133 Naples, Italy
[5] Department of Humanities, University of Naples Federico II, 80133 Naples, Italy
[6] Department of Biotechnological and Applied Clinical Sciences, University of L'Aquila, 67100 L'Aquila, Italy
[7] Institut de Neurosciences des Systèmes, Aix-Marseille Université, *13005* Marseille, France
[8] Department of Biomedical Sciences, University of Sassari, 07100 Sassari, Italy
* Correspondence: psorrentino@uniss.it
† These authors contributed equally.

The supplementary material includes detailed information regarding the processing pipeline and analysis of magnetoencephalography data reported in the manuscript. The following flow chart represents the main step of the data processing and analysis, in which the different colors refer to different steps: data preparation and filtering (blue), brain activity reconstruction or focusing (dark yellow), and connectome evaluation and statistical tests (green).

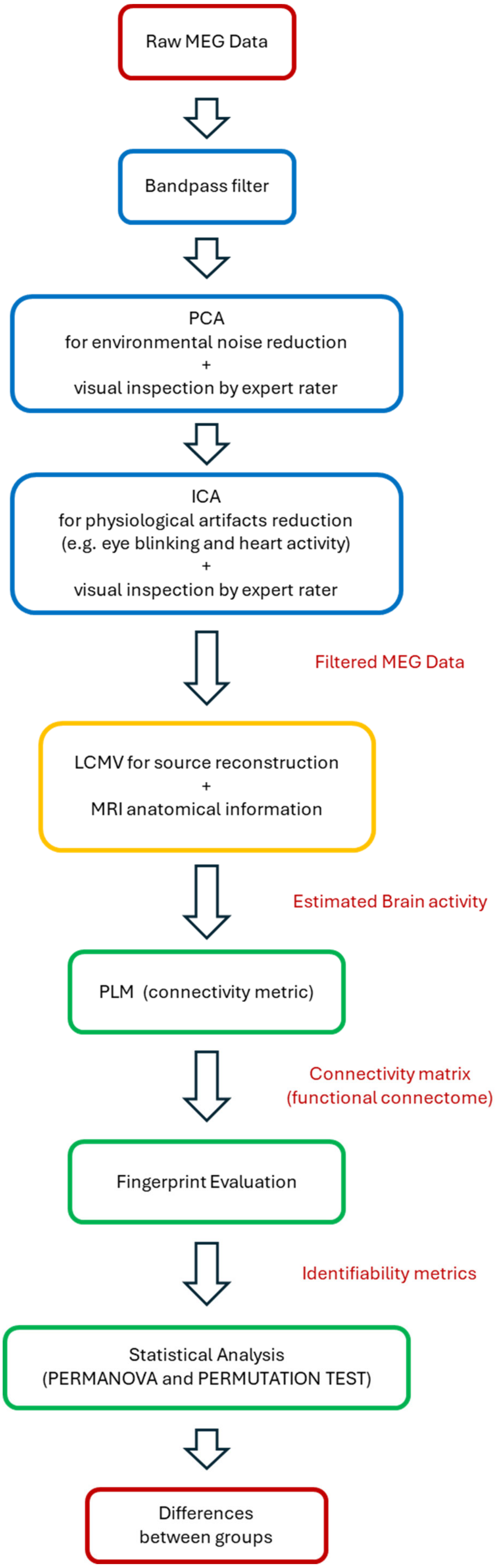
Raw MEG Data
Bandpass filter
PCA
for environmental noise reduction
+
visual inspection by expert rater
ICA
for physiological artifacts reduction
(e.g. eye blinking and heart activity)
+
visual inspection by expert rater
Filtered MEG Data
LCMV for source reconstruction
+
MRI anatomical information
Estimated Brain activity
PLM (connectivity metric)
Connectivity matrix
(functional connectome)
Fingerprint Evaluation
Identifiability metrics
Statistical Analysis
(PERMANOVA and PERMUTATION TEST)
Differences
between groups

BANDPASS FILTERING

The first step in the processing pipeline consists of data filtering. More in detail, a 4th order Butterworth IIR (Infinite Impulse Response) band-pass filter was adopted. For the sake of clarity, the following picture reports its magnitude (in dB) as function of the frequency:

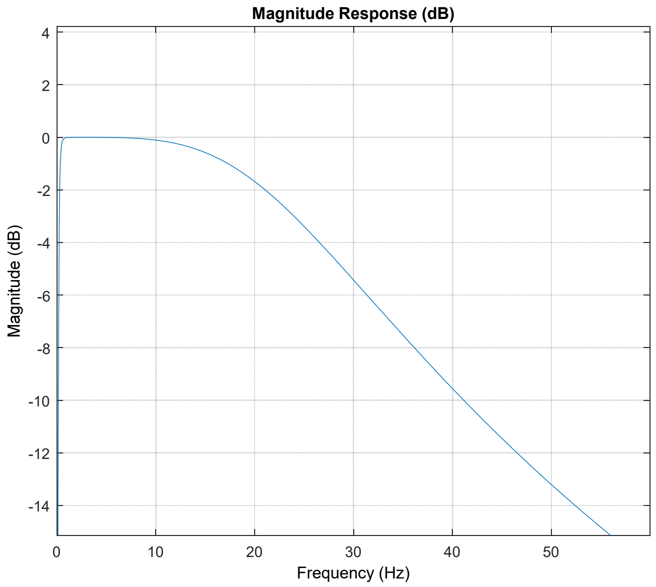

PRINCIPAL COMPONENT ANALYSIS (PCA)

Principal Component Analysis (PCA) is adopted to reduce the environmental noise. It is a statistical method used for dimensionality reduction and feature extraction. It works by transforming the original data into a new coordinate system (a set of orthogonal axes) such that the greatest variance by any projection of the data comes to lie on the first coordinate (called the first principal component), the second greatest variance on the second coordinate, and so on.

More in detail, the adopted PCA approach can be summarised in the following steps:

1. Centering the data
   Firstly, the mean of each signal collected by each channel (p in total) is subtracted to center the data around the origin. Thus, a matrix $\mathbf{X}$ containing all the collected signals organized in columns is obtained

2. Covariance matrix
   Then, the covariance matrix $\mathbf{\Sigma}$ of the data matrix $\mathbf{X}$ is computed. The covariance matrix between the features (i.e., columns) i and j is given by:
$$\mathrm{cov}(\mathbf{X}_i, \mathbf{X}_j) = \frac{1}{n-1} \cdot \sum_{k=1}^{n} (\mathbf{X}_{ki} - \overline{\mathbf{X}}_i)(\mathbf{X}_{kj} - \overline{\mathbf{X}}_j)$$
   where n is the number of samples of each signal, $\overline{\mathbf{X}}_i$ and $\overline{\mathbf{X}}_j$ are the means of the features i and j, respectively.
3. Eigenvalue decomposition
   After the previous steps, the eigenvalues $\lambda_1, \lambda_2, \dots, \lambda_p$ and the corresponding eigenvectors $\mathbf{v}_1, \mathbf{v}_2, \dots, \mathbf{v}_p$ of the covariance matrix Σ are calculated. The eigenvectors represent the directions of maximum variance, and the eigenvalues represent the magnitude along each eigenvector.

4. Principal components selection
The "principal components" are sorted in descending order of their corresponding eigenvalues. The first k principal components capture most of the variance in the data. In the considered case, k was chosen such that it retains 95% of the total variance.

5. Dimensionality reduction
Finally, the data is projected onto the subspace spanned by the first k principal components to reduce the dimensionality of data while preserving as much variance as possible.

INDEPENDENT COMPONENT ANALYSIS

Independent Component Analysis (ICA) was adopted to reduce physiological artifacts (i.e., eye blinking and heart activity). It is a computational technique for separating a multivariate signal into additive, independent components. It assumes that the observed multivariate data are linear mixtures of some underlying independent components. ICA aims to estimate these independent components by finding a linear transformation of the observed data that minimizes their statistical dependence.

More in detail, the adopted ICA can be summarized as:

1. Centering the Data
Similarly to PCA, ICA starts by centering the data so that each feature has zero mean.

2. Whitening the Data
ICA whitens the centered data by transforming it into a new space where the covariance matrix is the identity matrix. This decorrelates the data and ensures that all variables have unit variance.

3. Finding Independent Components
ICA then estimates the independent components by finding a linear transformation W that maximizes the statistical independence of the transformed signals. This is achieved by maximizing some measure of non-Gaussianity, such as negentropy or kurtosis.

4. Unmixing the Signals
Once the transformation matrix W is found, it is applied to the whitened data to obtain the estimated independent source signals.

LINEARLY CONSTRAINED MINIMUM VARIANCE BEAMFORMER

The Linearly Constrained Minimum Variance (LCMV) beamformer is a signal processing technique used in array processing, particularly in the field of sensor array processing and spatial filtering. It aims to spatially filter a set of observed signals to enhance the signal of interest while suppressing interference and noise, subject to linear constraints. The goal of LCMV beamforming is to find a set of weights (or spatial filter) that optimally combines the signals from the sensors to maximize the signal-to-noise ratio (SNR) of the output subject to linear constraints.

More in detail, the LCMV problem can be posed mathematically as:

$$\min_{\mathbf{W}(\mathbf{q})} \mathrm{tr}[\mathbf{W}^{\mathrm{T}}(\mathbf{q})\mathbf{C}(\mathbf{x})\mathbf{W}(\mathbf{q})] \quad \text{subject to} \quad \mathbf{W}^{\mathrm{T}}(\mathbf{q})\mathbf{L}(\mathbf{q}) = \mathbf{I},$$

in which the superscript "T" refers to the transpose operation, $\mathbf{W}$ is the weight matrix for the spatial filtering, $\mathbf{x}$ is the data collected at sensor locations (corrupted by noise), $\mathbf{C}(\mathbf{x})$ is the covariance matrix of the noisy data, $\mathbf{q}$ is a point of the considered volume, $\mathrm{tr}[\cdot]$ is the trace operation (performed on a matrix), $\mathbf{I}$ is the identity matrix and $\mathbf{L}$ is the matrix of the forward model relating the brain source activity with the signal collected at sensor locations (i.e., Leadfield matrix).

A solution to the previous problem can be obtained by using the method of Lagrange multipliers and completing the square, yielding to:

$$\mathbf{W}(\mathbf{q}) = [\mathbf{L}^T(\mathbf{q})\mathbf{C}^{-1}(\mathbf{x})\mathbf{L}(\mathbf{q})]^{-1}\mathbf{L}^T(\mathbf{q})\mathbf{C}^{-1}(\mathbf{x})$$

Finally, the previous equation and reminding that $\mathbf{y} = \mathbf{W}^T(\mathbf{q})\,\mathbf{x}$, it is possible to perform the source localisation by estimating the variance as a function of location withing the volume of interest.

PHASE LINEARITY MEASUREMENT

The Phase Linearity Measurement (PLM) is a metric to measure brain connectivity based on the analysis of similar behaviours in the phases of recorded signals. Its intrinsic characteristics include considerable noise rejection properties with respect to other widely adopted connectivity metrics.

The main aspect of this approach consists of its capability to identify signals which are synchronised in frequency with either a constant phase difference or a linear phase difference over time.

The main steps of the adopted PLM metric are reported in the following:

1. Computation of the analytic signals from the real-valued data.
2. Determination of the interferometric signal and its normalization.
3. Evaluation of the energy spectral density of the normalised interferometric signal $S_z(f)$.
4. PLM metric evaluation as ratio between energies, i.e.:

$$PLM = \frac{\int_{-B}^{B} S_z(f)\,df}{\int_{-\infty}^{\infty} S_z(f)\,df},$$

in which B represent the integration band (in our work, B = 1 Hz).

FINGERPRINT ANALYSIS

The fingerprint analysis based on the functional connectomes (FCs) is a methodology able to define subject-specific characteristics. Briefly, it starts from the definition of a matrix known as “Identifiability” or “Differentiation” matrix whose elements provide a measure of similarity by adopting the Pearson’s correlation between FCs of different subjects.

Person’s linear correlation coefficient is the most used linear correlation coefficient. For column $\mathbf{X}_a$ in matrix $\mathbf{X}$ and column $\mathbf{Y}_b$ in matrix $\mathbf{Y}$, having means $\bar{\mathbf{X}}_a = \frac{1}{n}\sum_{i=1}^{n}\mathbf{X}_{a,i}$, and $\bar{\mathbf{Y}}_b = \frac{1}{n}\sum_{j=1}^{n}\mathbf{Y}_{b,j}$, Pearson’s linear correlation coefficient $\rho(a,b)$ is defined as:

$$\rho(a,b) = \frac{\sum_{i=1}^{n}(\mathbf{X}_{a,i}-\bar{\mathbf{X}}_a)(\mathbf{Y}_{b,i}-\bar{\mathbf{Y}}_b)}{\left\{\sum_{i=1}^{n}(\mathbf{X}_{a,i}-\bar{\mathbf{X}}_a)^2\sum_{j=1}^{n}(\mathbf{Y}_{b,i}-\bar{\mathbf{Y}}_b)^2\right\}^{1/2}},$$

where n is the length of each column. Values of the correlation coefficient can range from -1 to +1. A value of -1 indicates perfect negative correlation, while a value of +1 indicates perfect positive correlation. A value of 0 indicates no correlation between the columns.

In our work, as described in Section “Fingerprint Analysis”, we adopted as metrics for measuring the similarity among subjects the I_{self}, I_{diff} and I_{others}.

STATISTICAL ANALYSIS - PERMANOVA

Permutational multivariate analysis of variance (PERMANOVA) is a non-parametric multivariate statistical permutation test. PERMANOVA is used to compare groups of objects and test the null hypothesis that the centroids and dispersion of the groups as defined by measure space are equivalent for all groups. A rejection of the null hypothesis means that either the centroid and/or the spread of the objects is different between the groups. Hence the test is based on the prior calculation of the distance between any two objects included in the experiment. PERMANOVA draws tests for significance by comparing the actual F test result to that gained from random permutations of the objects between the groups.

In the simple case of a single factor with p groups and n objects in each group, and $N = np$, the pseudo F-statistic is calculated as:

$$F = \frac{\left(\frac{SS_A}{p-1}\right)}{\left(\frac{SS_W}{N-p}\right)},$$

with:

$$SS_A = SS_T - SS_W, \qquad SS_T = \frac{1}{N}\sum_{i=1}^{N-1}\sum_{j=i+1}^{N} d_{ij}^2, \quad SS_W = \frac{1}{N}\sum_{i=1}^{N-1}\sum_{j=i+1}^{N} d_{ij}^2\,\delta_{ij},$$

and d_{ij}^2 being the squared distance between objects i and j, and δ_{ij} being 1 if the observations i and j belong to the same group, and 0 otherwise.

STATISTICAL ANALYSIS—PERMUTATION TEST

After that, a post-hoc analysis via a permutation test was performed to identify the pairs that significantly differ. The permutation test is based on random permutations of the data, which are used to create a null distribution for the test statistic.

1. Hypothesis formulation with a null hypothesis (H0) and an alternative hypothesis (H1).

2. Choice of a test statistic that measures the difference between the groups or conditions.

3. Calculation of the observed value of the test statistic using the original data.

4. Permutation Procedure:

    a. Pooling the data from all groups or conditions into a single dataset.
    b. Randomly shuffling the labels or group assignments while keeping the data values intact. This effectively breaks any relationship between the groups or conditions.
    c. Recalculation of the test statistic using the permuted data.

5. Repetition of the permutation process for a large number of times to create a distribution of the test statistic under the null hypothesis.

6. Comparing observed and permutated test statistics. If the observed test statistic is extreme (i.e., falls in the tails of the permutation distribution), it suggests that the null hypothesis is unlikely to be true.

7. Calculation of the p-value, which is the proportion of permuted test statistics that are as extreme as or more extreme than the observed test statistic. It represents the probability of observing such an extreme test statistic under the null hypothesis.

8. Make inference based on the p-value about whether to reject or fail to reject the null hypothesis.
